# Human-Inspired Holistic Control for Mobile Humanoid Robots

**DOI:** 10.3390/biomimetics11020130

**Published:** 2026-02-11

**Authors:** Zijian Wang, Xuanrui Ren, Hongfu Tang, Hongzhe Jin, Jie Zhao

**Affiliations:** School of Mechatronics Engineering, Harbin Institute of Technology, Harbin 150080, China; 24b308009@stu.hit.edu.cn (Z.W.); 25s008048@stu.hit.edu.cn (X.R.); tanghongfu@hit.edu.cn (H.T.); jzhao@hit.edu.cn (J.Z.)

**Keywords:** mobile manipulation, kinematics, whole-body motion planning and control

## Abstract

Humanoid mobile manipulators integrate a humanoid upper body with a mobile platform, forming a highly redundant system capable of performing complex manipulation tasks. To address the redundancy arising from the coordinated motion of the wheeled base, waist, and dual arms, this study proposes a human-inspired holistic control method based on multi-objective optimization. The degrees of freedom (DOF) of the upper limbs and the mobile base are unified within a single control framework, thereby enhancing overall motion coordination. Specifically, the controller is formulated as a strictly convex quadratic program (QP) that ensures accurate end-effector tracking while effectively handling joint position and velocity constraints. Inspired by human motor characteristics, the method incorporates a hierarchical weight assignment strategy and base DOF optimization to preserve arm manipulability while achieving effective coordination between the base and waist. Simulation studies of dual-arm handling tasks and real-world experiments involving mobile handling and peg-in-hole assembly demonstrate that the proposed method generates smooth, humanoid-like motions, thereby validating the effectiveness of the proposed control framework.

## 1. Introduction

Mobile humanoid robots, which combine a human-like upper body with a mobile platform, have attracted increasing attention due to their anthropomorphic morphology and potential [1] to perform multiple tasks in domestic and industrial environments [2,3,4]. Compared with fixed-base robotic arms, mobile platforms significantly expand the reachable workspace [5], enabling robots to execute tasks such as transportation, assembly, and service in large-scale workspaces. In particular, for mobile assembly and mobile handling tasks, robots can autonomously approach workpieces, grasp components, and transport them to target locations for precise assembly. Such capabilities are of critical importance in modern flexible manufacturing, warehouse logistics, and complex equipment maintenance. These tasks require not only large-range mobility but also accurate positioning and stable operation under base–arm coupling, highlighting the essential role of mobile control systems in real-world applications. Among various mobile configurations, wheeled bases have emerged as a mainstream solution [6] due to their high stability and efficiency. By avoiding the complex gait planning required for legged robots, wheeled platforms allow computational and mechanical resources to be more effectively allocated to manipulation performance [7].

Existing control strategies for mobile humanoid robots can generally be classified into two categories: force-based control methods and kinematic-based control methods.

Force control and optimization-based whole-body control (WBC) represent key approaches for achieving compliant behavior in redundant systems. These methods have been successfully applied in legged locomotion to maintain stability over complex and uneven terrains. For instance, Ref. [8] developed a compliance control framework for a large-scale quadruped robot, in which swing foot trajectories were optimized to enable safe traversal through narrow passages. Similarly, in the field of mobile manipulation, the mobile humanoid robot Rollin’ Justin employs a whole-body impedance-based controller to coordinate its dual arms, torso, and mobile base [9]. By incorporating whole-body dynamics into a hierarchical controller with strict task prioritization, compliant whole-body behavior is realized. In [10], a whole-body trajectory control framework was proposed based on a simplified dynamic model that accounts for both holonomic and nonholonomic constraints. However, wheeled humanoid robots constitute complex nonlinear systems, posing two major challenges for dynamic modeling: (1) the system complexity increases rapidly with the number of degrees of freedom, and (2) modeling accuracy is highly sensitive to parametric uncertainties and unmodeled dynamics. To mitigate these issues, advanced robust control strategies have been extensively explored to compensate for model inaccuracies. Notably, adaptive neural networks and sliding mode control techniques have been successfully applied to handle system uncertainties [11], and guarantee motion reliability under external disturbances [12]. Although Ref. [13] employed fuzzy control to address system uncertainties, its validation was restricted to low-dimensional task-space planning scenarios. In complex dynamic scenarios, such approaches may struggle to compute feasible trajectories in real time.

Kinematic control offers computational efficiency advantages for motion planning in mobile manipulators. Traditional approaches adopt a decoupled planning strategy, treating mobility and manipulation as separate subproblems. Ref. [14] proposed a pick-and-place framework that separates motion planning for the mobile base and manipulator, using a sampling-based planner for arm trajectory generation during grasping and Anytime Repairing A* (ARA*) for base navigation during transportation. However, such segmented planning often leads to poor motion continuity and reduced execution efficiency. To overcome these limitations, recent studies have focused on coordinated control strategies for integrated base–arm systems. Ref. [15] proposed a holistic planning algorithm based on the Jacobian pseudo-inverse, which optimizes manipulability while enforcing joint limit constraints at the acceleration level, and demonstrated its effectiveness in long-object transportation simulations. In [16], a virtual kinematic chain (VKC) was introduced for mobile dual-arm control, transforming complex dual-arm coordination into an equivalent single-chain motion problem. In this framework, the velocities of the mobile base and VKC joints are first optimized, and the resulting VKC motion is then mapped back to the joint velocities of the dual arms. In [17], a reactive whole-body control method was presented, formulating the motion controller as a Quadratic Programming (QP) problem that minimizes energy consumption while satisfying multiple constraints for efficient mobile manipulation. Building upon this work, a general reactive mobile manipulation framework was introduced in [18], explicitly accounting for base motion toward successive task objectives to improve grasping efficiency. Recently, Ref. [19] proposed an embodied holistic control for mobile manipulation (EHC-MM), which integrates Distant Mobility, Close Grasping (DMCG) principles into the QP-based formulation to coordinate the mobile base, manipulator, and onboard camera. Ref. [20] presented a whole-body differential inverse kinematics controller formulated within a QP framework, simultaneously addressing trajectory tracking, force regulation, obstacle avoidance, and singularity avoidance, with a task-specific heuristic control rate designed for pushing motions. In [21], a coordinated planning framework for mobile dual-arm robots was proposed based on manipulability optimization and force tracking, in which a hybrid control strategy was designed for the slave arm to integrate position control and force regulation, thereby satisfying heterogeneous task requirements in dual-arm manipulation.

Existing research have demonstrated that whole-body coordinated control is an effective means of improving the performance of mobile manipulation [22]. However, it is important to clarify the terminology used in this domain: while “whole-body control” generally refers to the physical utilization of all available redundant joints (base, waist, arms), “holistic control” emphasizes the mathematical integration of these degrees of freedom (DOFs) into a unified optimization formulation (e.g., a single QP), as opposed to employing decoupled control schemes for the base and manipulators.

Refs. [17,19,20] successfully established the holistic QP framework for mobile single-arm robots. Notably, Ref. [19] introduced the DMCG strategy, which coordinates the base and arm according to the distance to the target. While DMCG provides a robust heuristic for extending the workspace of a single arm, it is primarily a functional strategy designed for reachability. Such methods perform well in mobile manipulation tasks but are typically optimized for objects with small volume or low mass. In contrast, for target objects with large volume, high mass, or those requiring bilateral constraints, mobile dual-arm operation offers significant advantages in grasp stability, load sharing, and damage avoidance. This advantage is particularly evident in mobile handling and assembly tasks, where dual-arm systems better maintain object attitude stability and pose consistency during transportation and manipulation. However, for a humanoid dual-arm system equipped with a waist joint, coordination becomes substantially more complex than a simple distance-based switching between mobility and manipulation. Human motor control instead follows a proximal–distal coordination pattern, characterized by a continuous and hierarchical interaction among the arms, waist, and locomotion. Existing two-layer strategies (like DMCG) fail to fully capture this three-layer biological coordination involving the base, waist, and arms. Furthermore, in dual-arm tasks, the base must not only translate toward the target but also actively orient itself to satisfy the geometric symmetry required by bilateral arm coordination—a requirement explicitly addressed by the proposed biomimetic constraints but overlooked in existing distance-based frameworks.

**Figure 1:**
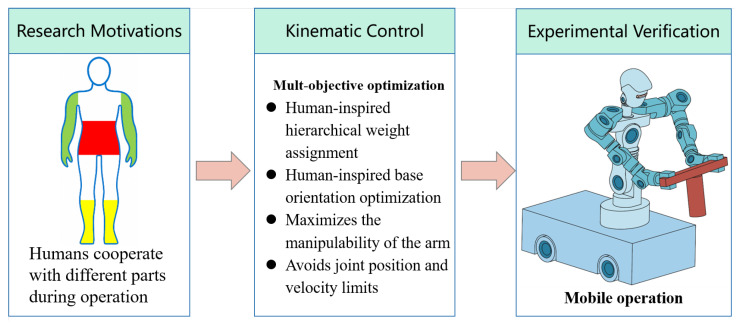
Overview of the proposed human-inspired holistic control framework for mobile humanoid robotic system.

To bridge the gap between generic holistic control and human-like dual-arm coordination, this paper proposes a human-inspired holistic control framework for mobile humanoid robots. The proposed method is formulated as a strictly convex quadratic programming (QP) problem within a multi-objective optimization framework. A hierarchical weighting strategy is introduced to prioritize the contributions of the arms, base, and waist, and a base orientation optimization method is designed to enhance arm coordination. Under a unified kinematic planning framework with joint constraints, smooth and coordinated whole-body motion is ensured throughout mobile manipulation tasks. An overview of the holistic control framework is highlighted in Figure 1.

The main contributions of this paper are summarized as follows.

1. A human-inspired holistic control method based on multi-objective optimization is proposed for mobile humanoid robots.
2. Inspired by human motor coordination principles, a hierarchical weight allocation strategy and base DOF optimization are developed to effectively coordinate the base and waist motions while preserving arm manipulability.
3. The effectiveness of the proposed method in enhancing whole-body coordination is validated through comparative simulations and real-world task execution experiments.

The remainder of this paper is organized as follows. Section 2 presents the kinematic modeling. Section 3 describes the design of the motion controller for the mobile humanoid robot. Section 4 reports the simulation results. Section 5 presents the experimental validation. Finally, Section 6 concludes the paper and discusses future research directions.

## 2. Kinematic Modeling

**Figure 2:**
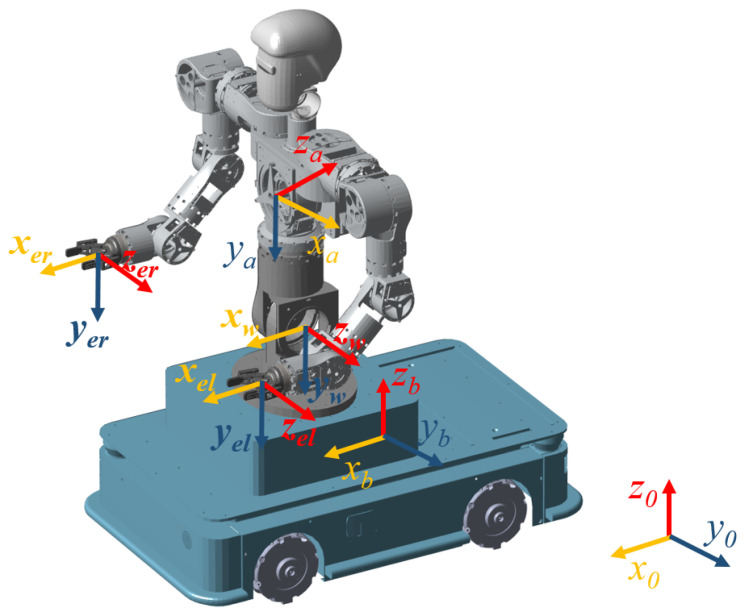
Coordinate establishment of mobile humanoid robot.

The mobile humanoid robot consists of two 8-DOF arms, a 3-DOF waist, and a 3-DOF omnidirectional Mecanum-wheeled base. Although the base physically supports full planar motion (translation in both axes and rotation), two distinct kinematic formulations are considered in this paper. First, to mimic human locomotor patterns, the base is modeled with a nonholonomic constraint (virtual differential drive), limiting the control inputs to forward translation and rotation. This approximation is particularly valid for tasks dominated by heading adjustments (e.g., centering the workspace), and serves as the primary model for the proposed human-inspired controller. Second, to demonstrate the generality of the framework, the kinematics are also extended to the full omnidirectional model. The kinematic structure is shown in Figure 2, and the detailed DH parameters can be found in [23].

### 2.1. Kinematics with Nonholonomic Constraints

The poses of the robot’s end-effectors are given by

(1) Tel0=Tb0(x,y,θ)TwbTaw(κw,qw)Tela(κl,ql)

(2) Ter0=Tb0(x,y,θ)TwbTaw(κw,qw)Tera(κr,qr)

where {0} denotes the world reference frame; Tb0 represents the pose of the mobile base {b} with respect to the world frame; and Twb is a fixed transformation from the mobile base frame to the base frame of the humanoid upper body. Taw denotes the forward kinematic mapping of the waist, where $\kappa_{w}$ corresponds to the waist kinematic parameters and $q_{w}$ denotes the waist joint configuration. Tela and Tera represent the forward kinematics of the left and right arms, respectively, with end-effector frames denoted by {el} and {er}. Here, $\kappa_{l}$ and $\kappa_{r}$ are the kinematic parameters of the two arms, and $q_{l}$ and $q_{r}$ are their corresponding joint coordinate vectors.

To incorporate planar base motion into the kinematic chain, the mobile base is modeled as virtual rotational and translational joints. An infinitesimal transformation is used to represent these two degrees of freedom, following [17].

(3) Twb=Tbb,(δθ,δd)Twb′

where $\delta_{\theta }$ and $\delta_{d}$ denote the infinitesimal rotation and forward translation of the nonholonomic base, respectively. The base can be modeled as a virtual revolute–prismatic mechanism with joint coordinates $q_{b}\in R^{2}$, where $∥q_{b}∥\to 0$, ${\dot{q}}_{b,0}={\dot{\delta }}_{\theta }$, and ${\dot{q}}_{b,1}={\dot{\delta }}_{d}$. Under this representation, the kinematic formulation becomes analogous to that of a serial-link manipulator. Following the approach in [24], the differential kinematics of the left manipulator can be expressed as

(4) vel0=Jel0(x,y,θ,qw,ql,qr)q˙bq˙wq˙l

which maps the velocity of joint of base, waist and left arm to the end-effector spatial velocity. The left manipulator Jacobian Jel0(·) is expressed in the world frame.

Similarly, the differential kinematics of the right arm can be derived. Since the left and right arms share common joints in the mobile base and the waist, designing their controllers independently may lead to conflicting motions of these shared components. Therefore, it is necessary to integrate the kinematics of both arms into a unified formulation.

(5) vel0ver0=Ja0Jela06×8Ja006×8Jeraq˙bq˙wq˙lq˙r

To simplify, we let

(6) ve0=vel0ver0

(7) Je0=Ja0Jela06×8Ja006×8Jera

(8) $$\dot{q}={\left({\dot{q}}_{b},{\dot{q}}_{w},{\dot{q}}_{l},{\dot{q}}_{r}\right)}^{⊤}$$

where ve0∈R12 denotes the end-effector space velocity of the left and right arms. The holistic manipulator Jacobian Je0∈R12×21 is expressed in the world frame. $\dot{q}\in R^{21}$ represents the joint velocity vector of the base, waist, and arms.

### 2.2. Extension to Omnidirectional Kinematics

Omnidirectional platforms are characterized by their ability to execute simultaneous translation and rotation within the 2D plane. By adopting the virtual joint formulation, the base configuration vector is defined as $q_{b}=\left(\delta_{x},\delta_{y},\delta_{\theta }\right)\in R^{3}$.

## 3. Mobile Humanoid Robot Motion Controller

In this paper, a QP-based motion controller is adopted to achieve whole-body motion control of the mobile humanoid robot. The controller is expressed as

**Figure 3:**
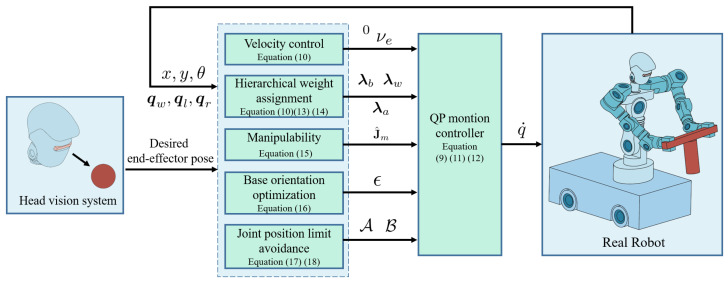
Block diagram of the mobile humanoid robot algorithm.

(9) minxfo(x)=12x⊤Qx+C⊤x,subjectto Je0x=0νeAx≤B

where $x=\dot{q}$ denotes the decision variable, *Q* is associated with the joint velocity cost, C incorporates manipulability maximisation and auxiliary performance objectives, and A and B enforce joint position limit avoidance as well as upper and lower bounds on the decision variables. The block diagram of the algorithm is represented as Figure 3.

### 3.1. Velocity Control

The end-effector has a spatial velocity

(10) ve0=Kpetrack+vr

where $e_{track}\in R^{12}$ represents the stacked tracking error for the dual arms computed on the SE(3) manifold. Let Tei0,Tei∗0∈SE(3) denote the current and desired poses of the *i*-th arm (where i∈{l,r} for left and right arms). The relative pose deviation is calculated as Ti=(Tei0)−1(Tei∗0). The error vector for a single arm $e_{tracki}={\left[e_{p,i}^{⊤},e_{o,i}^{⊤}\right]}^{⊤}\in R^{6}$ is defined as:

- **Position Error** $e_{p,i}\in R^{3}$: The translational component of $T_{i}$.
- **Orientation Error** $e_{o,i}\in R^{3}$: Calculated using the axis-angle representation $\theta_{i}k_{i}$, where $\theta_{i}$ and $k_{i}$ are the rotation angle and unit axis derived from the rotation matrix of $T_{i}$.

Finally, the total error is stacked as $e_{track}={\left[e_{l}^{⊤},e_{r}^{⊤}\right]}^{⊤}\in R^{12}$ to enable simultaneous coordination of both arms.

### 3.2. The Objective Function

The optimization problem minimizes the decision variable *x* in the objective function while satisfying the specified equality and inequality constraints. The objective function in (9) aims to minimize the joint velocity norm, maximize the robot manipulability as defined in [25], and incorporate auxiliary performance objectives. The objective function is formulated by

(11) $$Q=\mathrm{diag}\left(\lambda_{b},\lambda_{w},\lambda_{a}\right)\in R^{21\times 21}$$

(12) $$C=-{\hat{J}}_{m}+\epsilon \in R^{21}$$

where $\lambda_{b}$, $\lambda_{w}$, and $\lambda_{a}$ denote the weighting coefficients associated with minimizing the joint velocity norms of the base, waist, and arms, respectively. ${\hat{J}}_{m}$ represents the manipulability Jacobian corresponding to the arm joints [26]. Since the QP framework is formulated as a minimization problem, a negative sign is assigned to ${\hat{J}}_{m}$ so as to equivalently maximize the manipulability. Finally, ϵ represents the base orientation optimization term.

**Figure 4:**
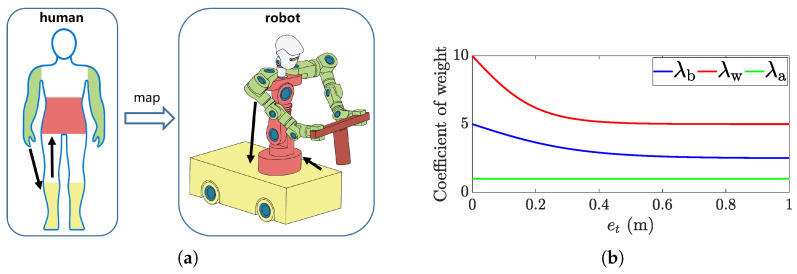
Hierarchical weight assignment: (**a**) Schematic diagram, simulation of human “first move the arm, then move the step, and finally adjust the waist” cooperative characteristics; (**b**) Weight coefficient curve.

*Human-inspired Hierarchical Weight Assignment*: A hierarchical weight allocation mechanism with human-like three-layer cooperation is proposed, reflecting the “proximal–distal” coordination strategy observed in human motion. Specifically, the arms are responsible for primary manipulation, the mobile base provides global mobility, and the waist contributes to postural stabilization. As illustrated in Figure 4a, the control priority is hierarchically assigned, decreasing from the arms to the base and further to the waist.

(13) $$\begin{array}{c} \lambda_{w}=\lambda_{w0}\left[1-\beta_{w}\cdot \sigma \left(e_{t},k_{w}\right)\right] \end{array}$$

(14) $$\begin{array}{c} \lambda_{b}=\lambda_{b0}\left[1-\beta_{b}\cdot \sigma \left(e_{t},k_{b}\right)\right] \end{array}$$

where σ(·) is a sigmoid response function defined as:

(15) $$\sigma \left(e_{t},k\right)=2\cdot \left(\frac{1}{1+e^{-k\cdot e_{t}}}-0.5\right)$$

Here, $e_{t}$ represents the accumulated position error magnitude used to trigger the adaptive mechanism. It is defined as the sum of the $L_{1}$ norms of the position deviations for both arms (i.e., $e_{t}={∥e_{pos,l}∥}_{1}+{∥e_{pos,r}∥}_{1}$), focusing on the reachability distance to regulate the base participation. This function maps the tracking error $e_{t}\in \left[0,\infty \right)$ to the range [0,1].

Two elements form this strategy:

1. Static weight initialization based on functional hierarchy: We assign $\lambda_{a0}=1$, $\lambda_{b0}=5$, and $\lambda_{w0}=10$. The rationale lies in balancing manipulability against stability. The arms ($\lambda_{a0}=1$) are assigned the lowest cost to serve as the primary execution units for precise manipulation. The mobile base ($\lambda_{b0}=5$) is penalized due to its large inertia, yet it is prioritized over the waist because it acts as the essential mechanism for expanding the workspace (Distant Mobility). The waist ($\lambda_{w0}=10$) is assigned the highest penalty. Although the waist aids in kinematic redundancy resolution, suppressing unnecessary waist motion is critical for maintaining upper-body stability. By assigning the highest weight, the controller ensures that the waist acts only as an auxiliary stabilizer, activated only when the arm-base coordination reaches its kinematic limits.

2. Error-adaptive adjustment: $\beta_{b}$, $\beta_{w}$ and $k_{b}$, $k_{w}$ govern the dynamic modulation based on the end-effector error $e_{t}$. The parameters $k_{b}=6$ and $k_{w}=10$ determine the sensitivity of the transition, ensuring a smooth but responsive adaptation, allowing the system to recruit the base and waist for error compensation when necessary. To ensure the stability of the QP solver, the weighting matrix *Q* must remain positive definite. In our implementation, we set $\beta_{b}=\beta_{w}=0.5$, ensuring that the dynamic weights are strictly lower-bounded (i.e., $\lambda \in [0.5\lambda_{0},\lambda_{0}]$). This prevents the weights from vanishing to zero regardless of the error magnitude. As illustrated in Figure 4b, as the error $e_{t}$ increases, the weights for the base and waist decrease significantly, narrowing the relative cost difference between these units and the arms. This reduction effectively elevates the participation priority of the base and waist, allowing them to be recruited for rapid, coarse positioning. Conversely, as $e_{t}$ decreases (indicating convergence), their weights rise to restore the strict hierarchy, suppressing gross body motion and shifting the control authority back to fine arm manipulation.

This mechanism enables a smooth transition from coarse approach to precise coordination during manipulation.

**Remark on Stability:** Although the proposed hierarchical weight assignment is formulated at the kinematic level and does not explicitly enforce dynamic stability criteria such as the Zero Moment Point (ZMP), it implicitly contributes to maintaining operational stability. By imposing a large penalty ($\lambda_{w0}$) on the waist joint, which carries significant mass—the controller effectively acts as a ‘virtual stiffness’ mechanism that restricts rapid and large-amplitude displacements of the trunk. This strategy limits the inertial forces generated by the upper body, thereby constraining the rate of change of the robot’s center of mass (CoM). Consequently, this mitigates the risk of structural oscillations and ensures that the system remains stable during standard mobile manipulation tasks.

*Manipulability*: The translational manipulability measure m(q) [25] is employed to evaluate the dexterity of the dual arms. Since the mobile base mainly contributes to large-scale positioning while the arms provide finer redundancy resolution, the manipulability gradient is formulated specifically with respect to the arm joints. The overall manipulability Jacobian ${\hat{J}}_{m}\in R^{21}$ used in the optimization cost is constructed as:

(16) $${\hat{J}}_{m}=(\begin{array}{cccc} 0_{b}, & 0_{w}, & J_{m}^{a}\left(q_{l}\right), & J_{m}^{a}\left(q_{r}\right) \end{array})$$

where $J_{m}^{a}\left(q_{l}\right)$ and $J_{m}^{a}\left(q_{r}\right)$ represent the gradients of manipulability with respect to the left and right arm joints, respectively. The waist joint is excluded due to its limited motion range and shared role between subsystems.

Explicit Computation of Gradient: Taking the left arm as an example, the translational manipulability is defined as $m_{lt}=\sqrt{\det(J_{lt}J_{lt}^{⊤})}$, where $J_{lt}\in R^{3\times 8}$ is the translational part of the arm Jacobian. The gradient $J_{m}^{a}\left(q_{l}\right)=\frac{\partial m_{lt}}{\partial q_{l}}$ is computed analytically to ensure online real-time performance.

Using the derivative property of the determinant, the *i*-th element of the gradient vector corresponds to the partial derivative with respect to the *i*-th joint $q_{i}$:

(17) $$\frac{\partial m_{lt}}{\partial q_{i}}=\frac{1}{2}m_{lt}\cdot \mathrm{tr}\left({\left(J_{lt}J_{lt}^{⊤}\right)}^{-1}\frac{\partial (J_{lt}J_{lt}^{⊤})}{\partial q_{i}}\right)$$

For efficient matrix implementation, this is reformulated using the Hessian tensor $H_{lt}\in R^{3\times 8\times 8}$ (where $H_{lti}=\frac{\partial J_{lt}}{\partial q_{i}}$):

(18) $$J_{m}^{a}{\left(q_{l}\right)}^{⊤}=m_{lt}\left(\begin{array}{c} \mathrm{tr}\left({\left(J_{lt}J_{lt}^{⊤}\right)}^{-1}J_{lt}H_{lt1}^{⊤}\right)\\ \vdots \\ \mathrm{tr}\left({\left(J_{lt}J_{lt}^{⊤}\right)}^{-1}J_{lt}H_{lt8}^{⊤}\right) \end{array}\right)$$

which can be compactly expressed using the vectorization operator vec(·) as:

(19) $${\left(J_{m}^{a}\left(q_{l}\right)\right)}_{i}=m_{lt}\cdot \mathrm{vec}{\left(J_{lt}H_{lti}^{⊤}\right)}^{⊤}\mathrm{vec}\left({\left(J_{lt}J_{lt}^{⊤}\right)}^{-1}\right)$$

In our implementation, the kinematic Jacobian $J_{lt}$ and the Hessian tensor $H_{lt}$ are computed analytically via standard geometric algorithms. This analytical formulation avoids numerical differentiation and is sufficiently computationally efficient to be executed within real-time control loops.

**Figure 5:**
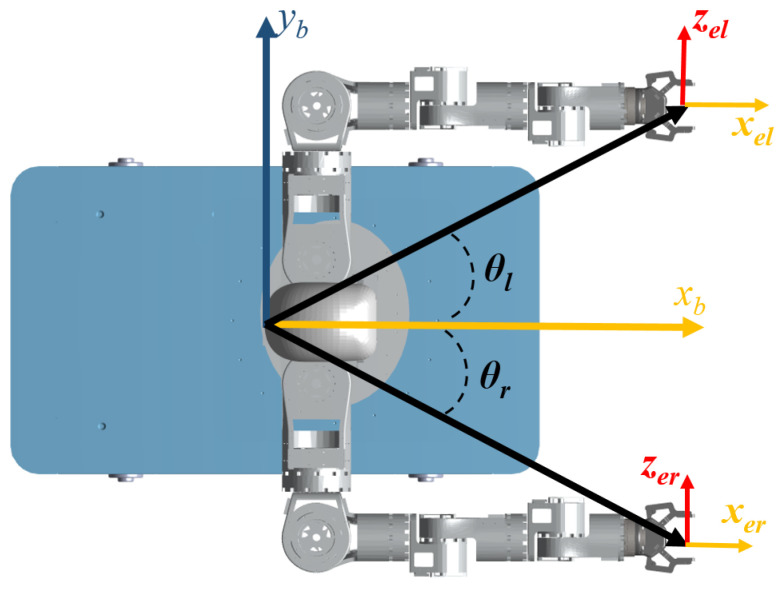
The motion controller makes the angles $\theta_{l}$ and $\theta_{r}$ of the base to the end-effectors of the two arms equal. This ensures high maneuverability of both arms.

*Human-inspired Base DOF Optimization*: As illustrated in Figure 5, the relative angles between the mobile base and the left and right end-effectors are denoted by $\theta_{l}$ (left) and $\theta_{r}$ (right). Inspired by the kinematic characteristics of human arms, which naturally operate on both sides of the waist, the optimal orientation of the robot base should lie along the symmetric midline between the two end-effectors; i.e., satisfying $\theta_{l}=-\theta_{r}$. In the mobile base coordinate frame {b}, the $x_{b}$ points forward along the robot’s heading, and the $y_{b}$ points to the left, so that $\theta_{l}$ is positive and $\theta_{r}$ is negative. To achieve this goal, the linear term in the cost function drives the base toward the median orientation of the two end-effector by optimizing the joint velocities, thereby enhancing the coordination of dual-arm operations. This cost is

(20) $$\epsilon =(-k_{\epsilon }\left(\theta_{l}+\theta_{r}\right),0_{1\times 20})$$

where θl=atan2(Tel1,3b,Tel0,3b), θr=atan2(Ter1,3b,Ter0,3b), and $k_{\epsilon }$ is a gain which adjusts how aggressively the base will be re-oriented. Specifically, $k_{\epsilon }$ is tuned to be sufficiently small (e.g., $k_{\epsilon }=5.0$ in our experiments) to ensure that this optimization term acts as a secondary objective. Since the end-effector tracking is enforced by strict constraints, this selection prevents the base re-orientation from conflicting with or dominating the primary manipulation task, allowing the robot to utilize redundancy for coordination without compromising tracking accuracy.

During dual-arm collaborative handling or mobile assembly tasks, this optimization term enhances the coordination of the dual-arm configuration, balances the load between both arms, improves overall manipulability and motion stability, and thereby increases the reliability of task execution.

### 3.3. Joint Position Limit Avoidance

Joint position-limit avoidance is enforced using velocity dampers, which constrain the robot’s joint velocities to slow down or prevent motion toward joint limits. No position-limit avoidance is applied to the virtual base joints. The velocity damper for joint position-limit avoidance is defined as

(21) $$A=\left(1_{21\times 21}\right)\in R^{\left(21\times 21\right)}$$

(22) $$B=\left(\begin{array}{c} 0_{b}\\ \eta \frac{\rho_{1}-\rho_{s}}{\rho_{i}-\rho_{s}}\\ \vdots \\ \eta \frac{\rho_{19}-\rho_{s}}{\rho_{i}-\rho_{s}} \end{array}\right)$$

where ρ represents the current distance to the closest joint limit, $\rho_{i}$ defines the range within which the damper begins to take effect, and $\rho_{s}$ denotes the minimum allowable distance from the joint to its limit.

## 4. Simulation Verification

### 4.1. Comparison Methods

In this chapter, the effectiveness of the proposed algorithm is verified by mobile handling simulation experiments. Two different types of algorithms are selected for comparative analysis to validate the superiority of the proposed framework from different perspectives.

**(1) Improved Clamping Weighted Least-Norm (ICWLN)**: First, the ICWLN [27] based on the pseudo-inverse method was selected as the benchmark for local kinematic control. The pseudo-inverse method is widely used in the industry because of its high calculation accuracy, strong robustness, and ease of engineering deployment. ICWLN optimizes the distribution of joint motion by introducing a weighting matrix, which can effectively realize the joint limit constraint and velocity constraint. Mobile humanoid robots possess ultra-high degrees of freedom, and their motion control faces unique challenges. Especially, excessive motion of the waist joint can easily lead to instability of the robot’s center of gravity, affecting deployment feasibility. Therefore, joint limit constraints must be rigorously considered. This is the primary reason for selecting ICWLN—its weighted optimization mechanism can effectively constrain the joint motion range, satisfying the stability requirements of humanoid robots.

**(2) Task Space Regions (TSR)**: Furthermore, the TSR framework [28] integrated with the Constrained Bi-Directional RRT (CBiRRT) planner is selected as a second baseline. This represents a typical global planning approach. By defining admissible regions for end-effector poses, TSR allows the planner to exploit the kinematic redundancy of the mobile manipulator to find collision-free paths in the configuration space.

### 4.2. Simulation Results

**Figure 6:**
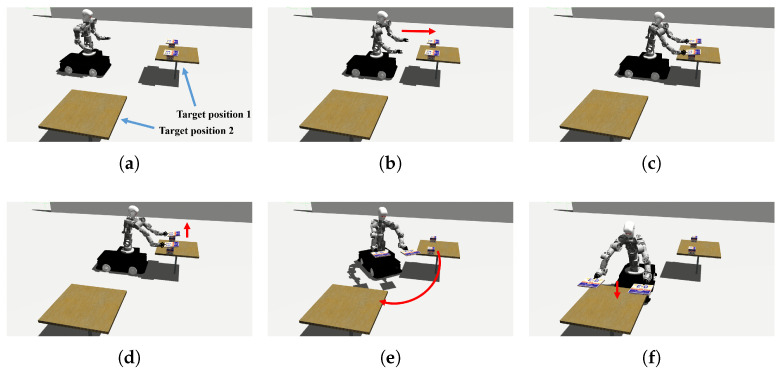
The handling process of the mobile dual-arm robot. (**a**) Preparation Phase; (**b**) Approach Phase; (**c**) Grasp Phase; (**d**) Lift Phase; (**e**) Handling Phase; and (**f**) Release Phase.

The simulation process is shown in Figure 6. The whole handling task is divided into six stages:

- Preparation Phase (0–5 s): The robot’s two arms move to the initial position.
- Approach Phase (5–10 s): The robot’s end-effectors move along the planned trajectory toward their respective grasping points.
- Grasp Phase (10–12 s): The grippers close to achieve stable and secure gripping of the target object.
- Lift Phase (12–17 s): The object is vertically lifted to a safe height to prevent collisions with the support surface.
- Handling Phase (17–32 s): The object is smoothly moved along the planned trajectory to the target position.
- Release Phase (32–34 s): The grippers open to deposit the object, completing the placement task.

### 4.3. Discussion of the Results

**Figure 7:**
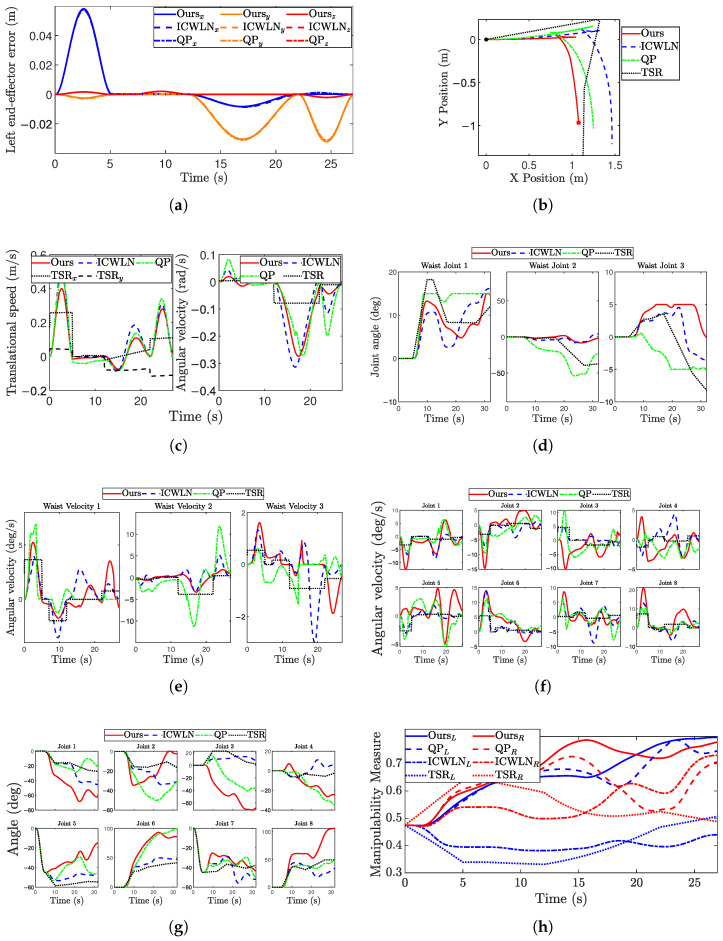
Simulation data of the mobile dual-arm robot handling process. (**a**) Left arm end error; (**b**) Robot base trajectories; (**c**) Base speed; (**d**) Waist joint angle; (**e**) Waist joint angular velocity; (**f**) Arm joint angular velocity; (**g**) Left arm joint angle; and (**h**) Manipulability of both arms.

The simulation results are presented in Figure 7. First, regarding trajectory tracking accuracy, the tracking error of the left end-effector remains below 0.06 m. The proposed method achieves comparable performance to ICWLN, confirming that the additional optimization term does not compromise the primary tracking task (Figure 7a).

Compared with ICWLN, the proposed method produces lower and more stable base velocities due to the hierarchical weight allocation (Figure 7c). For the waist joint (Figure 7d,e), the proposed approach generates smoother velocity distributions. This kinematic improvement is conducive to reducing the risk of upper-body vibrations and enhancing manipulation stability.

Distinct characteristics are also observed in the TSR baseline. While the TSR planner successfully completes the turn using base and waist rotation, as evidenced by the longest base trajectory in Figure 7b, and the motion quality is limited by its sampling-based nature. Since CBiRRT generates paths as discrete configurations, raw velocities derived from finite differencing suffer from discontinuities. Although post-processing smoothing was applied for a fair comparison, the velocity profiles of the base, waist, and arms (Figure 7c,e,f) still exhibit noticeable sawtooth-like fluctuations. This contrasts with the smooth, continuous motion generated by our QP-based framework, which is more favorable for minimizing mechanical wear and ensuring stability.

The arm joint trajectories (Figure 7g) of the proposed method exhibit slightly larger motion amplitudes compared to the baselines. This is because more posture adjustments are transferred to the upper body when the redundancy of the base and waist is constrained by our hierarchical strategy. This trend is consistent with human motion characteristics, where the arms contribute more active movement during precise manipulation phases.

To further quantify the effect of base DOF optimization, we analyzed the manipulability variations shown in Figure 7h. As observed in Figure 7h, the TSR baseline maintains a lower average manipulability compared to the proposed method. This is because standard sampling-based planners satisfy feasibility constraints but do not explicitly optimize instantaneous kinematic metrics, making the robot prone to near-singular configurations. In contrast, our proposed method achieves the highest average manipulability.

The average manipulability ($m_{avg}$) and average imbalance ($\Delta_{avg}$) are computed according to (23) and (24). The average imbalance ($\Delta_{avg}$) serves as a critical indicator of bimanual coordination quality. For independent dual-arm tasks, a low $\Delta_{avg}$ ensures motion synchrony. Crucially, for closed-chain cooperative tasks such as the assembly experiments in the next section, this metric becomes a critical factor for physical stability. A high imbalance suggests a “bottleneck” scenario where one arm approaches singularity while the other remains flexible, increasing the risk of internal geometric conflicts. By minimizing this imbalance, our method ensures that both arms maintain sufficient kinematic margins, providing a balanced capacity for load sharing.

Table 1 presents a comprehensive quantitative comparison. The proposed method achieves the highest average manipulability and the lowest imbalance. This quantitative improvement demonstrates that the base orientation optimization effectively prevents single-arm performance degradation, thereby enhancing both kinematic synchrony in simulation and operational stability in real-world applications. Beyond the average metrics, the minimum ($m_{min}$) and maximum ($m_{max}$) values provide further insights into the operational boundaries. The proposed method maintains a significantly higher $m_{min}$, ensuring a safer kinematic margin away from singular configurations throughout the trajectory. Furthermore, the highest Root Mean Square (RMS) value achieved by our method indicates a superior effective kinematic capacity. This demonstrates that the dual-arm system sustains a high level of dexterity and structural conditioning over the entire task duration, rather than merely peaking at specific instances.

(23) $$m_{avg}=\frac{1}{T}\sum_{t=1}^{T}\left(m_{l}\left(t\right)+m_{r}\left(t\right)\right)$$

(24) $$\Delta_{avg}=\frac{1}{T}\sum_{t=1}^{T}\left|m_{l}\left(t\right)-m_{r}\left(t\right)\right|$$

## 5. Experiment on Humanoid Robot

### 5.1. Experimental Setup

**Figure 8:**
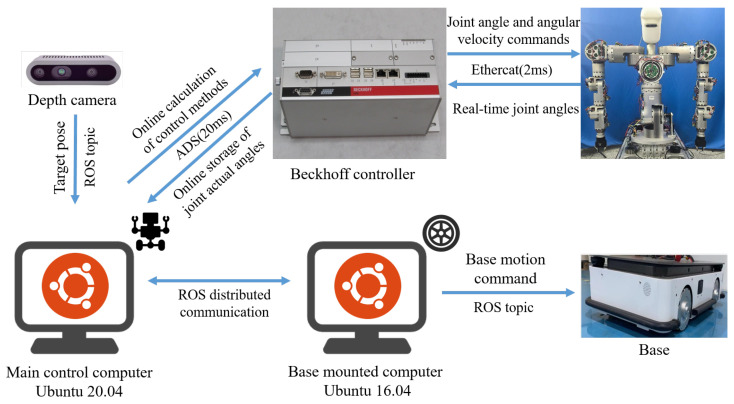
Experimental system.

The experimental setup, as illustrated in Figure 8, consists of a host computer (Intel Core i7) running Ubuntu 20.04, a Beckhoff controller, a humanoid upper-body robot, and a mobile base platform. The host computer handles real-time computation of control laws, records joint feedback, communicates with the base controller, and processes visual data from the depth camera. The Beckhoff controller receives joint angle and velocity commands from the host and drives the robot’s arm and waist accordingly. Simultaneously, the host publishes velocity commands to the mobile base through ROS topics.

The system operates at a control rate of 50 Hz. To validate the real-time feasibility of the proposed Human-inspired Holistic Control within this cycle, we analyzed the computational cost on the host PC. The controller is formulated as a QP problem with 21 decision variables and 33 linear constraints (12 task constraints and 21 joint limits). A fourth-order Runge–Kutta (RK4) method was employed for numerical integration, which requires the solver to execute four times per control step to ensure high-precision motion generation.

Performance analysis shows that for a continuous manipulation task of 37 s (1850 steps), the total computation time was only 393 ms, resulting in an average execution time of 0.21 ms per step. The OSQP solver demonstrated rapid convergence with an average of 25 iterations. Compared to the 20 ms control budget, the proposed framework occupies only 1% of the computational resources. This low computational load confirms that the algorithm is not only capable of the current 50 Hz offline planning but also possesses significant potential for high-frequency online deployment.

### 5.2. Results of Experiment

**Figure 9:**
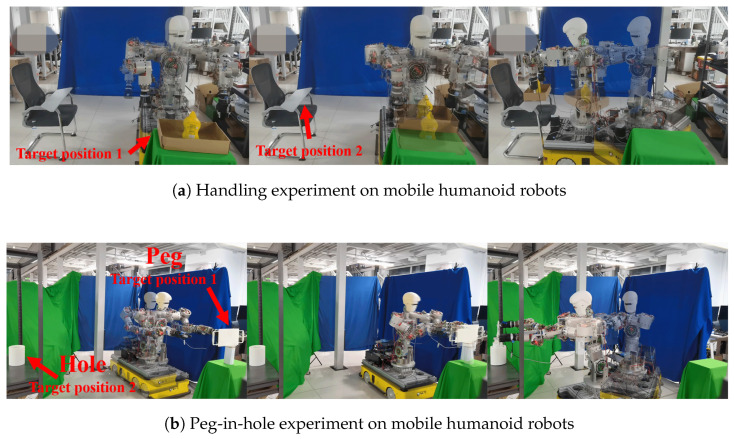
Experiment: (**a**) handling experiment, (**b**) peg-in-hole experiment.

To assess the effectiveness of the proposed algorithm, two experiments were performed on a mobile humanoid robot: a dual-arm object handling task and a peg-in-hole assembly task, as shown in Figure 9.

In the handling experiment (Figure 9a), the object was initially placed 1.5 m in front of the robot, and the target location was 1.3 m to its right. The entire process lasted 29 s, consisting of a 5 s preparation phase, a 5 s approaching phase, a 2 s grasping phase, a 5 s lifting phase, a 10 s transferring phase, and a 2 s releasing phase.

In the peg-in-hole experiment (Figure 9b), a T-shaped object was placed 1.6 m in front of the robot, with the target hole located 2 m to its right. The peg was a cylinder with a radius of 9 cm and a length of 18.5 cm. The task followed a similar sequence to the handling task, but with a 10 s approaching phase, a 15 s transfer phase, and a 5 s insertion phase, with an insertion depth of 16 cm.

### 5.3. Discussion of Experiment

**Figure 10:**
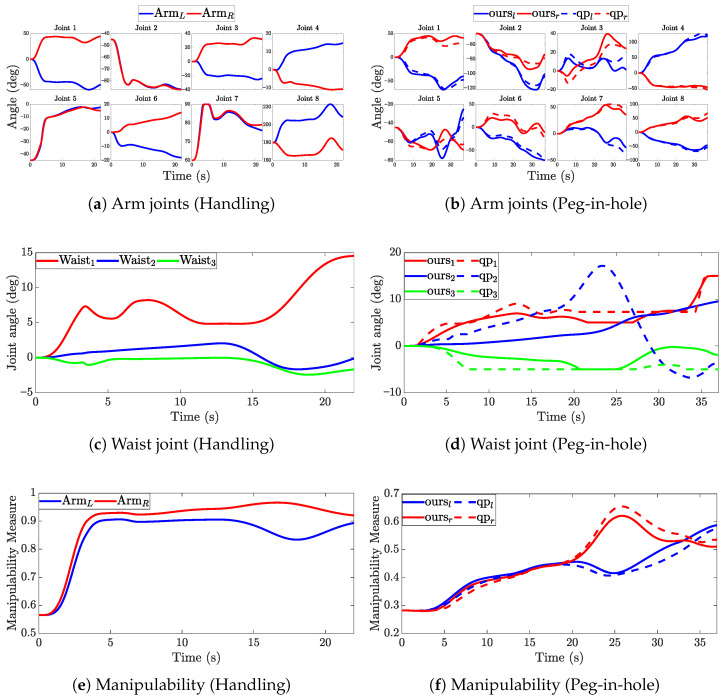
Experimental results showing joint trajectories and manipulability profiles. The left column (**a**,**c**,**e**) corresponds to the handling task, while the right column (**b**,**d**,**f**) corresponds to the Peg-in-hole task. The rows represent the arm joint angles, waist joint angle, and manipulability measure, respectively.

The results of the experiment are shown in Figure 10.

Figure 10a,c show the joint angle trajectories of the dual arms and the waist, respectively. All joint angles stayed within their admissible ranges, and the motions were smooth and continuous throughout the task. Figure 10e depicts the manipulability of the dual arms during the vertical grasping task, which remained around 0.9, indicating favorable postural manipulability.

In the assembly experiments, the proposed method was evaluated against a standard QP-based controller. Figure 10b shows the joint angle trajectories of both arms. In both experiments, all joint angles remained within their respective limits, and the motions were smooth and continuous throughout the task execution. Figure 10d presents the waist joint trajectories. It can be seen that the proposed method produces smaller waist joint excursions compared to the standard QP controller. This behavior is attributed to the hierarchical weight assignment strategy, which prioritizes arm motion for task execution while limiting excessive waist motion. Figure 10f illustrates the manipulability indices of both arms. Horizontal grasping results in lower manipulability values compared to vertical grasping. During the transportation phase, the right arm exhibits higher manipulability than the left arm. However, with the proposed base orientation optimization, the manipulability difference between the two arms is reduced. This indicates that the base optimization term effectively enhances bilateral coordination. During the insertion phase, the manipulability values of both methods converge to similar levels. Overall, the proposed method effectively reduces the manipulability imbalance between the two arms during transportation phases involving end-effector reorientation, which are critical for mobile assembly tasks.

These two sets of results demonstrate that the proposed algorithm successfully achieved stable whole-body motion control on the physical platform, thereby validating its effectiveness and reliability in practical applications.

**Summary**: This study successfully established a human-inspired whole-body control framework that effectively coordinates the mobile base, waist, and dual arms. By integrating hierarchical weight assignment with base orientation optimization, the proposed method significantly enhances manipulability and ensures smooth execution of complex mobile assembly tasks. However, the current scope focuses strictly on kinematic performance and does not yet consider active force feedback regulation. Consequently, the system relies primarily on geometric alignment and passive mechanical compliance. In scenarios with tight tolerances, initial misalignments (e.g., the axle contacting the hole’s rim) may pose challenges, as active compliance or local search behaviors (such as spiral search) are not currently implemented. Therefore, this work serves as a foundational kinematic alignment stage, paving the way for the future integration of impedance or admittance control to handle robust contact dynamics.

### 5.4. Extension to Omnidirectional Mobility

**Figure 11:**
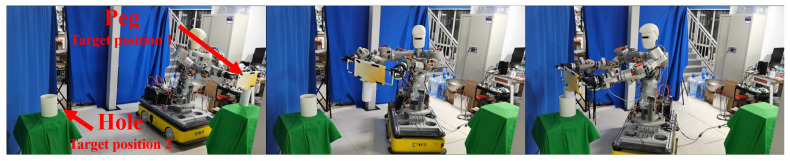
Peg-in-hole assembly experiment under the full 3-DOF omnidirectional mode.

To assess the generality of the proposed framework, the peg-in-hole assembly experiment was extended to the full 3-DOF omnidirectional mode. The experimental setup remained the same as described in Section 5.2, except that the robot’s base was allowed to move holonomically. Figure 11 shows the experimental process.

**Figure 12:**
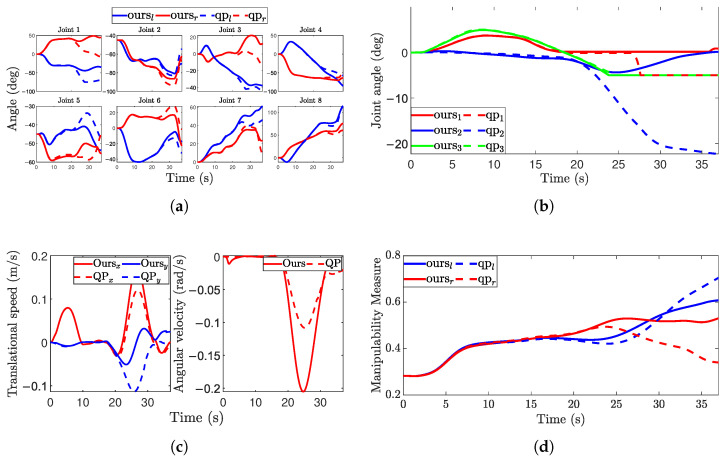
Data from the omnidirectional movement experiment. (**a**) Arm joints; (**b**) Waist joint; (**c**) Base speed; (**d**) Manipulability.

The comparative results between the proposed optimization method and the standard QP baseline on the 3-DOF base are presented in Figure 12. As observed in Figure 12c, the proposed method notably reduces the amplitude of translational motion while increasing the rotational component compared to the baseline. Visual inspection confirms that after the turning phase, the robot using our method maintains a forward-facing heading. In contrast, the standard QP baseline results in a side-facing orientation, causing a diagonal “strafing” motion. Moreover, the proposed method achieves a more coordinated dual-arm configuration. This is corroborated by the manipulability curves: as the turn progresses, the manipulability disparity between the two arms in the standard QP approach widens to 0.36, whereas our method maintains a minimal difference of just 0.071.

## 6. Conclusions

This study proposes a human-inspired holistic control algorithm for highly redundant mobile dual-arm robots. Within a multi-objective optimization framework, the upper limbs and the mobile base are uniformly formulated as a strictly convex quadratic programming (QP) problem, enabling integrated and coordinated whole-body control. By incorporating a human-inspired hierarchical weight assignment strategy together with base degree-of-freedom (DOF) optimization, the proposed method effectively coordinates the motions of the base and waist while preserving adequate arm manipulability. Both simulation and real-world experiments demonstrate that the proposed approach significantly improves whole-body motion coordination and motion smoothness in mobile manipulation tasks, thereby validating the effectiveness of the proposed control framework.

For future work, we aim to: (1) Integrate the proposed control framework with navigation and perception systems to enable mobile manipulation in complex and unstructured environments. (2) Incorporate force/torque feedback into the holistic control framework. While the current kinematic approach effectively achieves the geometric alignment required for assembly tasks with sufficient clearance, compliant behaviors based on impedance or admittance control are essential for tight-tolerance assembly phases; future work will therefore focus on explicitly addressing contact dynamics to ensure stability and prevent structural damage during demanding insertion tasks. (3) Extend the robot’s capabilities to more challenging tasks, such as the assembly of long-axis components. Such tasks require not only precise end-effector pose control but also a high level of whole-body coordination to maintain alignment accuracy during large-scale transportation motions.

## Figures and Tables

**Table 1:** Quantitative comparison of manipulability metrics across different control strategies. The best results are highlighted in bold.

Method	$m_{\mathrm{avg}}$	$\Delta_{\mathrm{avg}}$	$m_{\min}$	$m_{\max}$	RMS
**Proposed**	**1.3373**	**0.0421**	**0.9460**	**1.5768**	**1.3498**
Standard QP	1.2595	0.0568	**0.9460**	1.4545	1.2668
ICWLN	0.9653	0.1532	0.8803	1.1704	0.9683
TSR	0.9502	0.1579	0.8842	0.9945	0.9509

## Data Availability

The original contributions presented in this study are included in the article/Supplementary Materials.

## Supplementary Materials

The following supporting information can be downloaded at: https://www.mdpi.com/article/10.3390/biomimetics11020130/s1, Video S1: Human-Inspired Holistic Control for Mobile Humanoid Robots (Simulation and Experimental Demonstrations).
